# Digital Tools for the Recognition and Triage of Abnormal Uterine Bleeding: A Narrative Review

**DOI:** 10.7759/cureus.110510

**Published:** 2026-06-09

**Authors:** Hafsa Zahoor, Surbhi K Rajra

**Affiliations:** 1 Obstetrics and Gynecology, Acharya Shri Chander College of Medical Sciences and Hospital, Jammu, IND

**Keywords:** abnormal uterine bleeding, artificial intelligence, digital health, heavy menstrual bleeding, menstrual tracking, mobile health, patient triage, reproductive health, symptom checker, telemedicine

## Abstract

Abnormal uterine bleeding is a common gynaecological presentation, but patients frequently face difficulty distinguishing normal variation from bleeding that requires medical assessment. Digital health tools, including menstrual tracking applications, electronic bleeding diaries, telemedicine, eConsult systems, digital education, decision aids, and artificial intelligence-based risk prediction models, may improve recognition, documentation, triage, referral, and follow-up.

This narrative review synthesizes the available literature on digital tools relevant to abnormal uterine bleeding and heavy menstrual bleeding. A structured search of PubMed, Scopus, and Web of Science was conducted in May 2026 using terms for abnormal uterine bleeding, heavy menstrual bleeding, menstrual disorders, digital health, mobile applications, menstrual tracking, symptom checkers, telehealth, decision aids, patient portals, remote monitoring, artificial intelligence, and chatbots. After deduplication, 122 unique records were screened, and 27 articles were included.

The evidence shows that digital menstrual tracking can identify abnormal bleeding patterns at scale and may improve symptom histories, while mobile pictorial blood assessment charts and smartphone bleeding diaries offer more structured quantification than recall alone. Telemedicine and eConsult pathways appear feasible for selected abnormal uterine bleeding presentations when supported by safety-netting and clear thresholds for in-person assessment. Digital education and decision aids can improve knowledge and shared decision-making, although evidence for clinical outcomes is limited. Artificial intelligence models show promise for risk stratification, particularly for endometrial pathology, but require external validation, transparency, and clinical governance before routine deployment.

Overall, digital tools may support earlier recognition and more efficient triage of abnormal uterine bleeding, but current evidence remains fragmented. Future research should prioritize validated triage algorithms, patient-centred outcomes, equity, privacy, and integration with routine gynaecological workflows.

## Introduction and background

Abnormal uterine bleeding is a symptom-based clinical problem rather than a single diagnosis. It affects an estimated 10% to 30% of women of reproductive age, is the leading cause of iron-deficiency anaemia in this group, and reduces quality of life, while increasing the use of health services [1]. The 2018 revision of the International Federation of Gynecology and Obstetrics systems was intended to harmonize definitions of normal and abnormal uterine bleeding symptoms and classify potential causes of bleeding in the reproductive years [2].

Clinically, abnormal bleeding may involve changes in menstrual frequency, regularity, duration, volume, or intermenstrual bleeding. Acute presentations require prompt assessment for hypovolemia and hemodynamic instability, followed by classification of likely causes with the PALM-COEIN system after initial stabilization [3]. For non-acute presentations, effective care depends on a reliable history, recognition of red flags, timely investigation, and appropriate referral.

Despite this need, abnormal uterine bleeding is difficult to triage remotely because patients may normalize heavy or irregular bleeding, clinicians may lack structured information on bleeding pattern and volume, and urgent features such as pregnancy, postmenopausal bleeding, severe anaemia symptoms, or endometrial cancer risk may be missed. Digital tools have entered this gap in several forms. Menstrual tracking applications can capture longitudinal cycle and bleeding data. Electronic diaries and mobile pictorial blood assessment charts can standardize bleeding documentation. Telemedicine and eConsult systems can direct patients toward urgent, routine, or procedural care. Digital education and decision aids can support shared decision-making, while artificial intelligence may assist risk stratification. Practical guidance has already described how telemedicine can be used for abnormal uterine bleeding evaluation and management, but the wider evidence base remains dispersed across different digital technologies and clinical populations [4].

This review aims to synthesize evidence on digital tools that support recognition, documentation, triage, referral, and follow-up for abnormal uterine bleeding and heavy menstrual bleeding. The review focuses on clinical utility, implementation, safety, equity, privacy, and research gaps relevant to routine gynaecological care.

## Review

Methodology

A structured narrative review was conducted. PubMed, Scopus, and Web of Science were searched in May 2026. The search combined terms for abnormal uterine bleeding, heavy menstrual bleeding, menorrhagia, intermenstrual bleeding, menstrual disorders, and menstrual bleeding with terms for digital health, mobile health, mobile applications, period trackers, menstrual tracking, symptom checkers, telehealth, telemedicine, decision aids, patient portals, remote monitoring, artificial intelligence, and chatbots. Records were exported from each database, combined, and deduplicated before screening.

Articles were eligible when they addressed a digital tool relevant to recognition, documentation, self-assessment, triage, referral, education, shared decision-making, remote care, or risk stratification for abnormal uterine bleeding or heavy menstrual bleeding. Eligible designs included primary studies, qualitative studies, development studies, randomized trials, cohort analyses using app-generated data, telemedicine studies, eConsult evaluations, digital education studies, decision-aid studies, and relevant reviews. Articles were excluded when they focused only on pharmacological or surgical treatment, pathology after tissue sampling, vaccine-associated menstrual changes, fertility tracking without bleeding assessment, or digital health in unrelated clinical domains.

The database search yielded 230 records. After deduplication, 122 unique records were screened. Twenty-seven articles met the inclusion criteria and were synthesized narratively. Because included studies differed substantially in population, tool type, outcomes, and study design, meta-analysis was not appropriate. Findings were organized by digital function into six categories that map onto the steps of the abnormal uterine bleeding care pathway, from recognition and documentation through triage, referral, and risk stratification: longitudinal menstrual tracking, bleeding quantification, education and decision support, telemedicine and referral pathways, artificial intelligence-based risk stratification, and digital patient experience. Within each category, findings on clinical utility, feasibility, implementation, and limitations were synthesized narratively.

**Figure 1:**
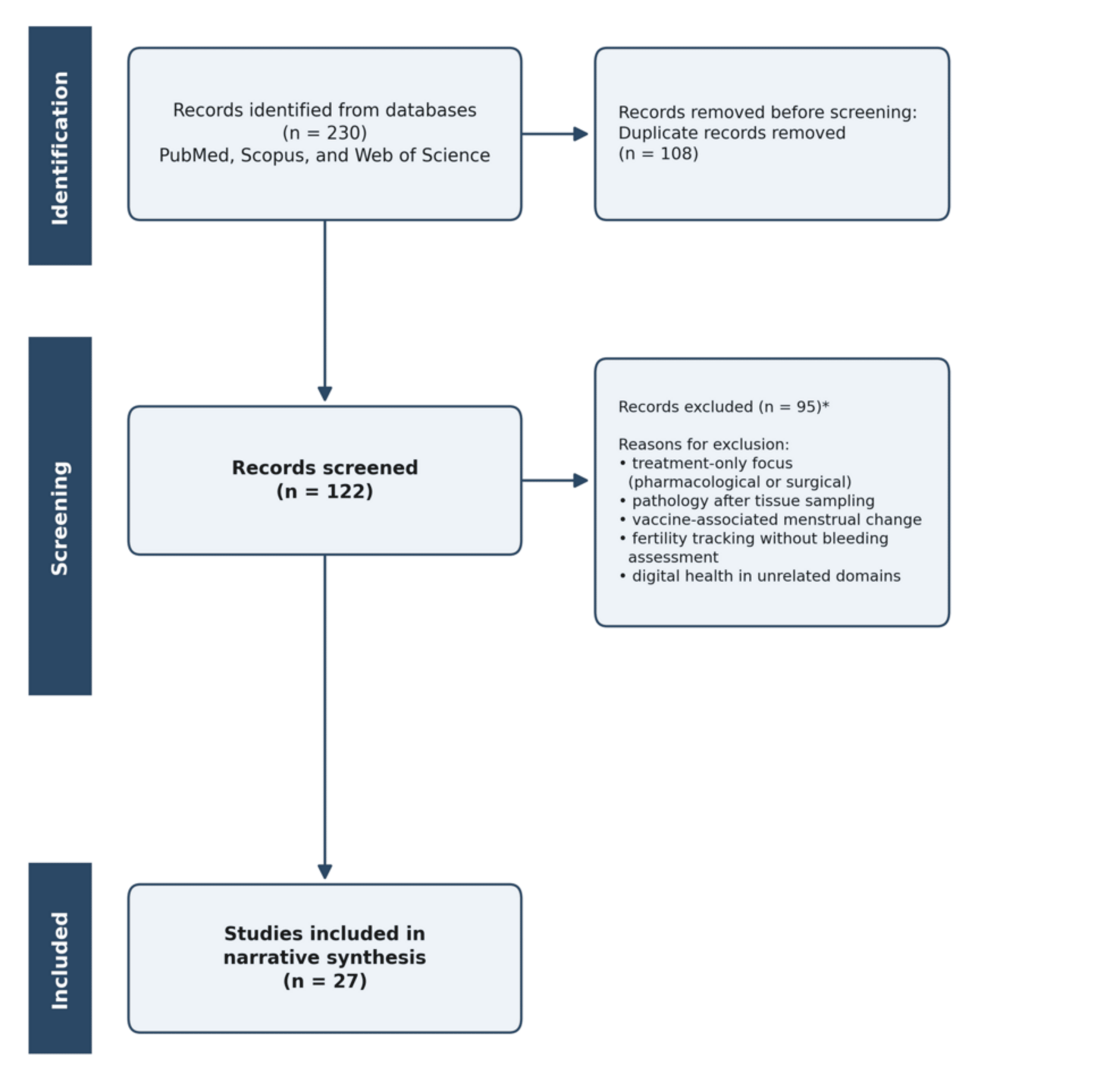
PRISMA flow diagram showing identification, screening, and inclusion of studies in the narrative review

Figure 1 provides a PRISMA flow diagram showing the identification, screening, and inclusion of studies in the narrative review.

Digital menstrual tracking and recognition of abnormal bleeding patterns

Menstrual tracking applications are the most visible digital tools related to abnormal uterine bleeding, but their clinical value depends on whether they collect interpretable data and whether patients and clinicians can use those data safely. A qualitative study of patient and provider perspectives found that menstrual apps can support recording of symptoms and menstrual disorders, but also highlighted the need for better clinical integration and interpretation [5]. This point is important because raw cycle logs may not automatically translate into a clinically meaningful abnormal uterine bleeding assessment.

The Apple Women’s Health Study provides one of the strongest examples of how digital cohort design can support menstrual health research at scale. Its methods paper described a digital longitudinal cohort with mobile collection of menstrual cycle and health data [6]. A subsequent analysis used menstrual tracking to identify abnormal uterine bleeding patterns among participants, demonstrating that app-derived cycle data can be used to characterize bleeding abnormalities in a large population [7]. Shea et al. further showed that app tracking reveals variability in the construct of heavy menstrual bleeding, emphasizing that bleeding burden is not captured fully by one symptom or one threshold [8]. Together, these studies suggest that digital tracking can improve recognition and epidemiological understanding of abnormal bleeding, but they also show that symptom definitions and user-entered data need careful interpretation.

Large app-based datasets also provide contextual information beyond cycle timing. A digital cohort study of menstrual product use in the United States found that product-use patterns vary by sociodemographic, health, and menstrual characteristics, information that could improve how clinicians interpret bleeding histories and product saturation reports [9]. A mobile application-based cross-sectional study among Chinese women of reproductive age similarly used app data to describe menstrual patterns and disorders [10]. In adolescents, mobile app measurement has been used to capture menstrual cycle characteristics, dysmenorrhea, and activity limitation in real time [11]. Sustained engagement and usability are particularly important in this age group, and a recent adolescent menstrual-cycle app study examined whether young users remained engaged over time [12]. The overall implication is that tracking tools can extend the history beyond recall, but data completeness, health literacy, language, privacy, and sustained use determine whether these tools can support triage.

Digital measurement of bleeding burden

For triage, bleeding volume is often harder to assess than timing. Clinicians commonly ask about pad or tampon changes, flooding, clots, anaemia symptoms, and interference with school, work, or daily life. Digital pictorial tools and smartphone diaries may make this history more structured. In a randomized crossover study, a mobile application was compared with a paper pictorial blood assessment chart for tracking menses in young women, showing that mobile formats can be used to collect bleeding information in a structured way [13]. Su et al. used a WeChat QR code approach with pictorial blood assessment and menstrual attitude measures to assess heavy menstrual bleeding prevalence and knowledge among gynaecology outpatients, illustrating how familiar digital platforms can be used for screening and education in clinical populations [14].

Bleeding diaries also matter in contexts where bleeding is a treatment effect or adverse outcome. A comparison of paper diaries, text messages, and smartphone app tracking in contraceptive studies found that digital methods can collect bleeding and symptom data, although each method has usability and data-quality considerations [15]. These studies do not prove that digital bleeding diaries improve outcomes for abnormal uterine bleeding, but they show that structured digital capture is feasible. For clinical triage, the key opportunity is to convert self-reported bleeding into actionable categories such as urgent assessment, routine workup, medical treatment review, or procedural planning. This requires validated thresholds and explicit safety-netting rather than passive tracking alone.

Table 1 summarizes tool functions, rather than endorsing any specific commercial application.

**Table 1:** Digital tool categories relevant to abnormal uterine bleeding triage

Digital tool category	Potential role in abnormal uterine bleeding care	Representative evidence	Key limitation
Menstrual tracking applications	Longitudinal cycle and bleeding documentation; recognition of abnormal patterns	Provider and patient perspectives; Apple Women’s Health Study; app-based menstrual disorder datasets [5-10]	Self-entered data may be incomplete, inconsistently coded, or difficult to interpret clinically
Mobile bleeding quantification tools	Structured assessment of bleeding burden using mobile pictorial charts or diaries	Mobile pictorial blood assessment chart, WeChat survey, and digital diary comparisons [13-15]	Thresholds for triage and referral require validation across populations
Digital education and decision aids	Improves knowledge, prepares users for care-seeking, and supports preference-sensitive decisions	Electronic intervention, WeThrive app, digital education review, shared decision-making review, and MENTIP trial [16-20]	Education does not guarantee access, diagnostic completion, or treatment uptake
Telemedicine and eConsult pathways	Remote history-taking, safety-netting, referral prioritization, and preparation for investigations or procedures	Telemedicine guidance, paediatric and adolescent gynaecology visits, eConsult studies, see-and-treat model, and pandemic data [21-25]	Not all presentations can be managed remotely; urgent features require in-person assessment
Artificial intelligence and prediction tools	Risk stratification for endometrial pathology and prediction of treatment-related bleeding patterns	Data mining, artificial intelligence cancer-risk models, technology-enabled cancer interception, and intrauterine system companion app [26-29]	External validation, explainability, bias testing, and governance are required
Online forums and peer-support platforms	Reveals patient concerns, help-seeking barriers, and unmet informational needs	Thematic analysis of heavy menstrual bleeding discussions in online forums [30]	Peer information may be variable in accuracy and may not direct users to timely clinical care

Digital education, decision aids, and shared decision-making

Recognition of abnormal bleeding is not purely a measurement problem. It is also an education and communication problem. Digital interventions may help users understand when bleeding is outside expected limits, when pregnancy testing is needed, when anaemia symptoms should prompt care, and why postmenopausal bleeding requires evaluation. An electronic intervention for young women with heavy menstrual bleeding showed that electronic approaches can be used to reach adolescents and young adults with targeted information [16]. The WeThrive app, designed for adolescents who menstruate, used a mobile health format to address heavy menstrual bleeding, and connect users to local clinics, and qualitative findings suggested that adolescents valued accessible, relevant information and connection to care [17].

A systematic review of female digital health education found that digital educational interventions are being used across women’s health conditions, including conditions that can present with chronic abnormal uterine bleeding [18]. For heavy menstrual bleeding specifically, decision support has a longer history than mobile apps. A systematic review of shared decision-making interventions for heavy menstrual bleeding found a small evidence base and emphasized the need for tools that support preference-sensitive choices [19]. The MENTIP randomized controlled trial tested a computerized decision aid in primary care for menorrhagia and evaluated its effects on decision-making and quality of life [20]. These studies support the idea that digital triage should not simply tell patients whether to seek care. It should also prepare them for shared decisions about investigation, medical treatment, intrauterine systems, procedural options, and fertility goals.

Telemedicine, eConsult, and remote care pathways

Telemedicine is the digital modality most directly connected to triage. During the coronavirus disease 2019 pandemic, expert guidance and rapid review evidence addressed telemedicine for outpatient gynaecological scenarios, including abnormal uterine bleeding [21]. Paediatric and adolescent gynaecology experience also suggests that telemedicine can be used for selected visits, including abnormal uterine bleeding, while identifying patients who require in-person follow-up [22]. These findings are consistent with the central safety principle that abnormal uterine bleeding can be initially assessed remotely in many cases, but not all presentations can be completed remotely.

Electronic consultation systems add another layer by supporting clinician-to-clinician triage. A gynaecology eConsult service at a quaternary academic medical centre included abnormal uterine bleeding among common consultation reasons and showed how asynchronous specialist input can improve access and guide next steps [23]. A patient-centred see-and-treat model for abnormal uterine bleeding proposed restructuring the consultation so that preliminary workup and counselling can occur before an in-person procedure-oriented visit [24]. In Brazil, the coronavirus disease 2019 pandemic affected the diagnosis and treatment of abnormal uterine bleeding, and telehealth-based consultation data highlighted both the usefulness and limitations of remote care during disrupted health services [25].

The practical role of telemedicine in abnormal uterine bleeding is therefore best understood as a front-end clinical organization. Remote history-taking can identify emergency features, pregnancy possibility, anticoagulant use, postmenopausal bleeding, anaemia symptoms, and risk factors for endometrial pathology. It can also prepare for laboratory testing, imaging, medication counselling, or procedural planning. However, triage pathways must define clear thresholds for urgent in-person assessment, pelvic examination, ultrasound, endometrial sampling, or emergency care. Without such thresholds, telemedicine may create false reassurance or delay diagnosis.

Artificial intelligence and risk stratification

Artificial intelligence has a narrower but potentially important role in abnormal uterine bleeding triage. The most relevant models focus on identifying the risk of endometrial intraepithelial neoplasia or endometrial cancer among people with bleeding symptoms. Farzaneh et al. evaluated data mining classification methods for endometrial cancer in women with abnormal uterine bleeding, demonstrating how clinical variables can be used in predictive models [26]. Erdemoglu et al. developed artificial intelligence models to predict endometrial intraepithelial neoplasia and endometrial cancer risk in premenopausal and postmenopausal women, incorporating bleeding status and clinical variables [27]. DeStephano et al. discussed opportunities to expand access to endometrial cancer interception using technologies such as smartphone applications, artificial intelligence, gamification, and patient education [28].

These studies support a future in which digital intake forms or patient portals could flag higher-risk abnormal uterine bleeding before specialist review. For example, age, menopausal status, body mass index, polycystic ovary syndrome, diabetes, medication exposure, family history, and bleeding pattern could be combined to prioritize investigation. However, algorithmic triage is safety-critical. Models trained in one population may not generalize to another; missing data can distort predictions; and opaque risk scores may be difficult for clinicians to audit. Artificial intelligence should therefore be used as decision support only. External validation, calibration, explainability, bias testing, and governance are essential before such tools are used to direct urgency or defer care.

Digital prediction is also relevant outside of cancer risk. A digital medical device companion for new intrauterine system users was developed to support counselling, including artificial intelligence-based bleeding-pattern prediction after device initiation [29]. This shows how predictive tools may help patients understand expected bleeding changes and reduce anxiety, but such tools should distinguish expected treatment-related changes from abnormal symptoms that require reassessment.

Patient experience, online help-seeking, equity, and privacy

Digital triage begins before a patient reaches a clinic. Many people search online, use forums, or compare experiences with peers before seeking medical advice. A thematic analysis of online forum discussions found that heavy menstrual bleeding users sought validation, practical support, and information while describing disruption to daily functioning and frustration with medical dismissal [30]. This evidence is important because it shows why patient-facing tools must be empathetic, clear, and action-oriented. A technically accurate tool that does not acknowledge stigma, embarrassment, fear, work disruption, or prior dismissal may not change help-seeking behaviour.

Equity is a recurring concern across digital abnormal uterine bleeding tools. App-based studies can overrepresent users with smartphones, health literacy, English-language access, and comfort with tracking intimate data. Telemedicine can reduce travel and waiting time, but it can also exclude patients with poor internet access, limited private space, low digital literacy, disability-related barriers, or language needs. Algorithms may also reproduce bias if they are trained on datasets that underrepresent adolescents, older patients, racialized populations, rural communities, or people with complex comorbidities. Privacy is especially important because menstrual and reproductive data are sensitive. Users should know what data is collected, how it is stored, whether it is shared, and whether it can be deleted. Clinicians should be cautious about recommending commercial apps without understanding their privacy practices and clinical limitations.

Evidence gaps and future research priorities

The current evidence base supports feasibility more strongly than outcomes. The literature shows that people can record menstrual data digitally, complete mobile bleeding assessments, receive electronic education, use decision aids, attend telemedicine visits, and be triaged through eConsult or predictive models. However, few studies directly test whether these tools reduce diagnostic delay, improve appropriate referral, detect anaemia earlier, increase completion of endometrial evaluation when indicated, reduce emergency attendance, improve quality of life, or improve patient trust. In addition, studies use heterogeneous definitions and outcomes, making synthesis difficult.

Future research should move from tool development to pathway evaluation. Studies should test whether digital abnormal uterine bleeding triage improves patient-centred and clinical outcomes compared with usual care. Outcomes should include early and reliable diagnosis of cases of abnormal uterine bleeding, time to first clinical contact, time to ultrasound or biopsy when indicated, anaemia diagnosis, emergency visits, treatment initiation, procedural readiness, patient understanding, decisional conflict, privacy concerns, and equity of access. Digital tools should be validated in adolescents, reproductive-aged adults, perimenopausal patients, and postmenopausal patients because risk thresholds and clinical priorities differ across groups. Implementation studies should also evaluate how digital data appear in the electronic record, who reviews alerts, how urgent messages are escalated, and how clinicians manage liability when patient-entered data are incomplete or inaccurate.

Table 2 identifies gaps that should be addressed before digital abnormal uterine bleeding triage is adopted widely.

**Table 2:** Evidence gaps and future research priorities

Evidence gap	Why it matters	Suggested research direction
Lack of validated abnormal uterine bleeding triage algorithms	Digital tracking alone does not determine urgency or diagnostic need	Prospective validation of algorithms against clinician assessment, urgent outcomes, anaemia, imaging, and endometrial pathology
Limited patient-centred outcome data	Feasibility does not prove that tools improve care	Measure time to care, diagnostic completion, decisional conflict, quality of life, treatment initiation, and patient trust
Weak integration with clinical workflows	Patient-generated data are useful only if reviewed and acted on safely	Implementation studies of portals, alerts, clinician dashboards, escalation workflows, and documentation burden
Equity and access concerns	Digital tools may exclude people with lower digital literacy, privacy constraints, or poor connectivity	Recruit diverse populations and report outcomes by age, language, socioeconomic status, geography, disability, and race or ethnicity where appropriate
Privacy and governance uncertainty	Menstrual and reproductive data are sensitive and may be collected by commercial platforms	Evaluate consent, data sharing, retention, deletion, cybersecurity, and clinician responsibility before recommending tools

The certainty of this evidence also varies by study design. The strongest evidence comes from a small number of higher-quality sources, including the MENTIP randomized controlled trial of a computerized decision aid [20] and large digital cohort studies such as the Apple Women's Health Study [6,7], which show that decision support and app-based bleeding data can be studied rigorously and at scale. Much of the remaining literature consists of feasibility, development, and qualitative studies, or single-platform and single-centre evaluations, which are useful for demonstrating acceptability but are more preliminary and often of limited generalizability. Several relevant studies also address technologies adjacent to abnormal uterine bleeding rather than dedicated triage tools. The conclusions of this review should therefore be weighted accordingly, with greater confidence in feasibility than in clinical effectiveness.

Limitations of this review

This review has limitations. It was designed as a structured narrative review rather than a systematic review with duplicate independent screening, formal risk-of-bias assessment, or meta-analysis. The search used three major databases, but relevant app evaluations, commercial product documentation, implementation reports, or non-English studies may have been missed. Because the search was conducted in May 2026, and because digital health, artificial intelligence-based tools, and menstrual health applications are evolving rapidly, the review reflects the evidence available at that time and is likely to become outdated as new studies appear. The evidence base was heterogeneous, and several included articles addressed menstrual tracking, education, telemedicine, or risk prediction adjacent to abnormal uterine bleeding rather than testing a dedicated abnormal uterine bleeding triage tool. Therefore, the conclusions should be interpreted as a synthesis of an emerging field rather than evidence that any specific digital triage system is ready for universal clinical adoption.

## Conclusions

Digital tools can support abnormal uterine bleeding care by improving recognition, structuring symptom documentation, enabling remote triage, preparing patients for shared decision-making, and helping clinicians prioritize risk. Menstrual tracking applications and digital cohort studies show that bleeding patterns can be captured at scale. Mobile bleeding assessment tools and electronic diaries can make bleeding histories more structured. Telemedicine and eConsult pathways can improve front-end organization of care when they include clear escalation criteria. Artificial intelligence models may eventually support risk stratification for endometrial pathology.

The strongest message from the current literature is that digital tools should augment, not replace, clinical judgment. Abnormal uterine bleeding includes low-risk, chronic, and urgent presentations, and triage tools must be designed around safety, equity, privacy, and workflow integration. Future research should evaluate complete digital care pathways rather than isolated applications, using the patient-centred and clinical outcomes set out above.
